# The prognostic effect of sixteen malnutrition/inflammation-based indicators on the overall survival of chemotherapy patients

**DOI:** 10.3389/fimmu.2023.1117232

**Published:** 2023-02-16

**Authors:** Tong Liu, Chenan Liu, Li Deng, Mengmeng Song, Shiqi Lin, Hanping Shi

**Affiliations:** ^1^ Department of Gastrointestinal Surgery/Clinical Nutrition, Capital Medical University Affiliated Beijing Shijitan Hospital, Beijing, China; ^2^ Beijing International Science and Technology Cooperation Base for Cancer Metabolism and Nutrition, Beijing, China; ^3^ Key Laboratory of Cancer Food for Special Medical Purposes (FSMP) for State Market Regulation, Beijing, China; ^4^ Institute of Gastroenterology, The First Hospital of Nanchang, Nanchang, China

**Keywords:** cancer, chemotherapy, indicators, inflammation, lymphocyte-to-CRP ratio

## Abstract

**Background:**

Studies have confirmed the validity of malnutrition/inflammation-based indicators among cancer patients compared to chemotherapy patients. Moreover, it is necessary to identify which indicator is the best prognostic predictor for chemotherapy patients. This study attempted to determine the best nutrition/inflammation-based indicator of overall survival (OS) for chemotherapy patients.

**Methods:**

In this prospective cohort study, we collected 16 nutrition/inflammation-based indicators among 3,833 chemotherapy patients. The maximally selected rank statistics were used to calculate the optimal values of cutoffs for continuous indicators. OS was evaluated using the Kaplan–Meier method. The associations of 16 indicators with survival were evaluated using Cox proportional hazard models. The predictive ability of 16 indicators was assessed *via* time-dependent receiver operating characteristic curves (time-ROC) and the C-index.

**Results:**

All indicators were significantly associated with worse OS of chemotherapy patients in the multivariate analyses (all P < 0.05). Time-AUC and C-index analyses indicated that the lymphocyte-to-CRP (LCR) ratio (C-index: 0.658) had the best predictive ability for OS in chemotherapy patients. The tumor stage significantly modified the association between inflammatory status and worse survival outcomes (P for interaction < 0.05). Compared to patients with high LCR and I/II tumor stages, patients with low LCR and III/IV tumor stages had a 6-fold higher risk of death.

**Conclusions:**

The LCR has the best predictive value in chemotherapy patients compared with other nutrition/inflammation-based indicators.

**Clinical trial registration:**

http://www.chictr.org.cn, identifier ChiCTR1800020329.

## Introduction

Cancer is the leading cause of mortality globally. An estimated 19.3 million new cancer cases and nearly 10 million cancer-related deaths are expected in 2020. With an estimated 2.3 million cases, female breast cancer has surpassed lung cancer as the most diagnosed cancer, followed by lung cancer (11.4%), colorectal cancer (10.0%), and prostate cancer (10.0%) (1). Cancer mortality, which has been increasing during most of the 20th century, continued to decline from its peak in 1991 to 2018, with a total decline of 31% due to the reduction in smoking and improvements in early detection and treatment in the United States (2). However, a significant change is taking place in China’s cancer profile, with more cancers that were previously more prevalent in the United States now being diagnosed in China. Since 2000, China has seen a gradual increase in cancer cases and deaths, as well as crude incidence and mortality rates (3).

The bidirectional relationship between inflammation and cancer was reported previously. Inflammation is a critical component of tumor initiation and progression. In addition, it is thought that tumor cells use innate immune system signals to invade, migrate, and metastasize, including selectins, chemokines, and their receptors (4). Chemotherapy is one of the most commonly used treatments for the majority of cancers. As chemotherapy evolves, its role will be expanded further and will play an even greater role in improving cancer patient survival and quality of life (5). However, there are concerns about the side effects of chemotherapy, despite its benefits for cancer patients. First, chemotherapy has been found to increase the levels of local inflammation (6). Additionally, inflammation contributes to a poor chemotherapy response and shorter survival rates in cancer patients than in those without inflammation (7). Second, acute and late toxicities of chemotherapy, nausea, vomiting, diarrhea, and mucositis further lead to malnutrition and even cachexia. In short, nutrition/inflammation-related factors can be used as effective prognostic predictors for cancer patients receiving chemotherapy. The prognostic effect of several nutrition/inflammation-related factors on the overall survival of cancer patients has been validated previously, including C-reactive protein (CRP), platelet-to-lymphocyte ratio (PLR), neutrophil-to-lymphocyte ratio (NLR), advanced lung cancer inflammation index (ALI), systemic immune-inflammation index (SII), CRP/albumin ratio (CAR), albumin-to-globulin ratio (AGR), modified Glasgow prognostic score (mGPS), geriatric nutritional risk index (GNRI), nutritional risk index (NRI), prognostic nutritional index (PNI), controlling nutritional status (CONUT) score, glucose-to-lymphocyte ratio (GLR), lymphocyte-to-CRP ratio (LCR), lymphocyte CRP score (LCS), and modified GNRI (mGNRI).

Predicting of prognosis for cancer patients receiving chemotherapy is challenging; hence, validated biomarkers for prediction of patient survival are urgently needed to help identify patients and administer timely and effective treatment. Although studies have confirmed the validity of the aforementioned indicators among cancer patients, whether similar results could be extrapolated to cancer patients receiving chemotherapy is unclear. Moreover, it is necessary to identify which indicators are the best prognostic predictors for chemotherapy patients. In the current study, we evaluated and compared 16 malnutrition/inflammation-based biomarkers for their predictive and prognostic role in OS in chemotherapy patients.

## Materials and methods

### Study population

**Figure 1:**
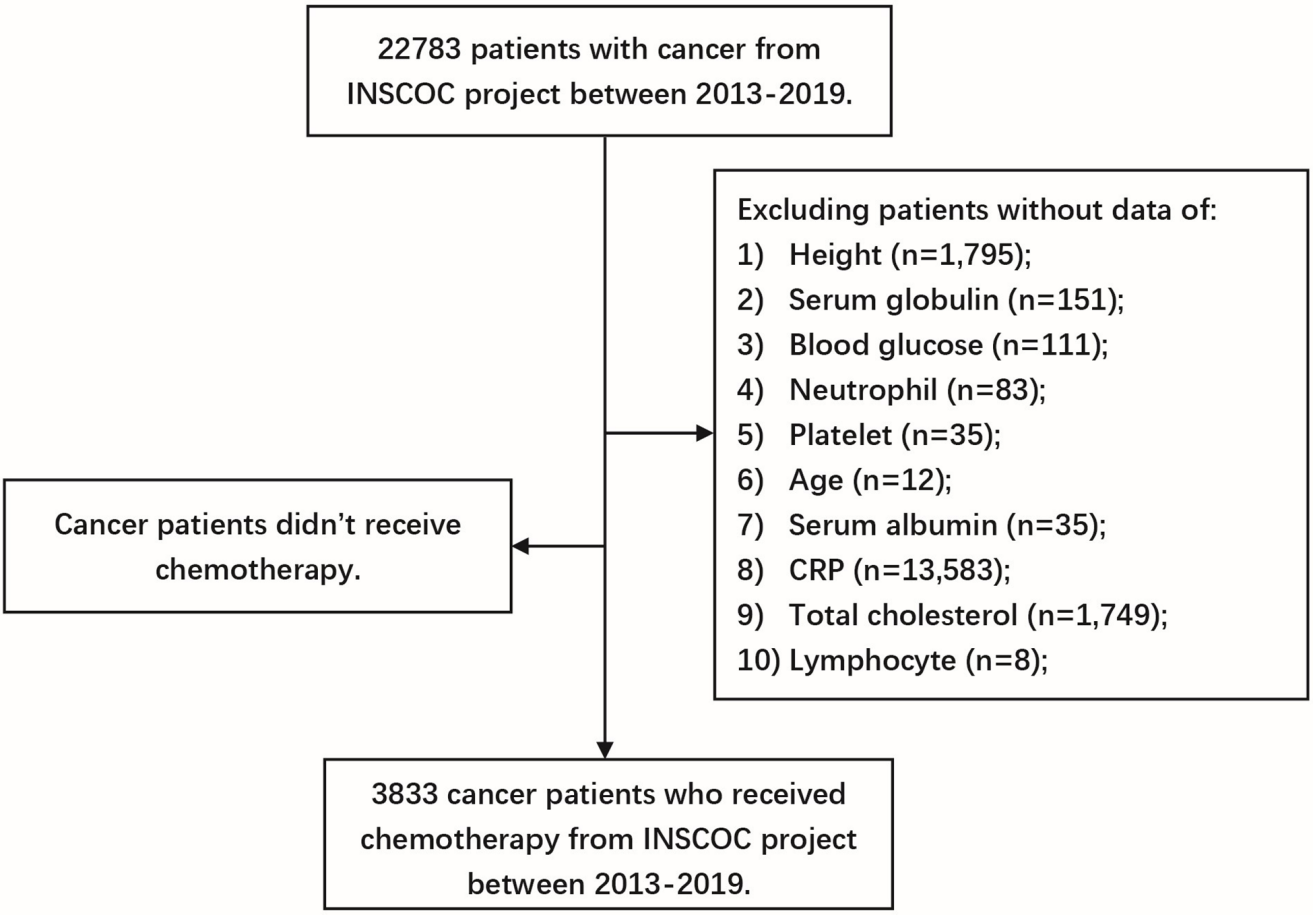
The detailed enrolled procedure of eligible patients in the INSCOC project from 2013-2019.

Participants were drawn from the Investigation on Nutrition Status and its Clinical Outcome of Common Cancers (INSCOC) project (http://www.chictr.org.cn, registration number ChiCTR1800020329), which is an ongoing, multicenter, prospective cohort study. The study design, methods, and development were described previously (8, 9). In short, all patients with a pathologically diagnosed malignancy were between 18 and 90 years of age at the time of admission. Patients must have no communication impairment, and be able to complete the study questionnaire. In addition, patients must be willing to participate in the study and be able to provide informed consent. In the current study, we excluded participants without data on 16 malnutrition/inflammation-based biomarkers in the current study. We also excluded participants who did not receive chemotherapy in INSCOC. A total of 3,833 cancer patients with all data for 16 malnutrition/inflammation-based biomarkers and who received chemotherapy were included in the final analysis (**Figure 1**). Informed consent forms were signed by all patients or their representative relatives within 48 h of hospital admission and before study initiation. According to the Declaration of Helsinki, the study protocol was approved by the institutional review board of Beijing Shijitan Hospital.

### Baseline characteristics collection

Data on socioeconomic factors and lifestyle behaviors of the patients were collected by trained medical staff *via* a standard questionnaire. In addition, all patients underwent routine assessment by dietitians to acquire nutritional information and data on anthropometric measurements, including height, body weight, handgrip strength, Patient-Generated Subjective Global Assessment (PG-SGA) score, and Karnofsky Performance Score (KPS). Tumor stage was evaluated using the 8th edition of the American Joint Committee on Cancer TNM staging system and was classified as stage I, II, III, and IV.

### Measurements of nutrition/inflammation-based indicators

Within 24 hours of hospitalization, routine blood examinations, including total protein, albumin, total cholesterol, glucose, hemoglobin, globulin, creatinine, triglyceride, CRP, total cholesterol, neutrophil, lymphocyte, red blood cells, and platelet counts, were conducted for all patients upon admission of the first time. In the current study, the nutrition/inflammation-based indicators included CRP, PLR, NLR, ALI, SII, CAR, AGR, mGPS, GNRI, NRI, PNI, CONUT, GLR, LCR, LCS, and mGNRI. The classifications of LCS, CONUT, and mGPS were based on previous studies. **Table S1** shows the current study’s detailed calculation methods for all nutrition and inflammation-based indicators.

### Statistical analysis

All statistical computations were performed using SAS software (version 9.4) and R software, version 3.4.3 (https://www.r-project.org). Variables were expressed as the mean ± standard deviation, median (interquartile range), or absolute number with the proportion as appropriate for the continuous or categorical variables. Based on the optimal cutoff points calculated using maximally selected rank statistics, we dichotomized the continuous nutrition/inflammation-based indicators. Kaplan−Meier curves were used to evaluate overall survival (OS), and log-rank tests were performed to analyze the differences. The association between nutrition/inflammation-based indicators and overall survival was evaluated using the Cox proportional hazard model. C-indices and time-dependent receiver operating characteristic curves (time-ROCs) were used to assess the predictive accuracy of each indicator. The three best indicators were selected for further analysis. Net reclassification improvement (NRI) and integrated discrimination improvement (IDI) were used to measure the improvement in the risk prediction model performance for the top three indicators. Moreover, possible modifications of the associations among the three best indicators and outcomes were assessed for variables including sex, median age (<59 vs. >59 years), tumor stage (I/II vs. III vs. IV), smoking status (none vs. past/current), drinking status (no vs. past/current), and BMI (<18.5 vs. 18.5-24 vs. ≥24 kg/m^2^). All analyses were statistically significant at p-value < 0.05 (two-sided).

## Results

### Baseline characteristics of the study population

The mean age of the patients was 58.22 ± 10.71 years, with 2,202 (57.4%) men and 1631 (42.6%) women. **Table 1** shows the baseline clinicopathological characteristics of cancer patients who received chemotherapy in the INSCOC project from 2013 to 2019. Lung cancer (34.7%) was the most common cancer type, followed by colorectal cancer (19.5%), gastric cancer (13.5%), and breast cancer (10.9%). Nearly half of the patients (47.5%) had stage IV tumors. A total of 46.6% and 22.6% of patients consumed tobacco or alcoholic beverages, respectively. A total of 55.7% of patients received surgery for cancer, and 16.9% of 3,833 cancer patients who received chemotherapy received radiotherapy in the current study.

**Table 1:** Baseline clinicopathological characteristics of cancer patients who received chemotherapy in the INSCOC project from 2013-2019.

Variables		Overall
Age (mean (SD))		58.22 (10.71)
Gender (%)	Men	2202 (57.4)
	Women	1631 (42.6)
BMI (mean (SD))		22.38 (3.14)
Tumor types (%)	Lung cancer	1330 (34.7)
	Gastric cancer	519 (13.5)
	Breast cancer	418 (10.9)
	Colorectal cancer	748 (19.5)
	Other types	818 (21.3)
Tumor stage (%)	I	241 (6.8)
	II	608 (17.2)
	III	1008 (28.5)
	IV	1679 (47.5)
Current/past smoker (%)		1785 (46.6)
Current/past drinker (%)		865 (22.6)
Receive radiotherapy (%)		646 (16.9)
Receive targeted therapy (%)		184 (4.8)
Receive immunotherapy (%)		154 (4.0)
Receive surgery (%)		2136 (55.7)
History of cirrhosis or hepatitis (%)		163 (4.3)
History of stroke (%)		20 (0.5)
History of CVD (%)		154 (4.0)
Hypertension (%)		751 (19.6)
Diabetes (%)		369 (9.6)
Total protein (mean (SD))		68.76 (6.58)
Albumin (mean (SD))		39.16 (4.85)
Total cholesterol (mean (SD))		4.65 (1.11)
Blood glucose (mean (SD))		5.74 (1.71)
Hemoglobin (mean (SD))		120.56 (28.23)
Serum creatinine (median [IQR])		64.90 [55.00, 76.80]
Triglyceride (median [IQR])		1.27 [0.95, 1.76]
CRP (median [IQR])		3.70 [2.89, 15.30]
Neutrophil count (median [IQR])		3.55 [2.51, 5.09]
Lymphocyte count (median [IQR])		1.50 [1.10, 1.91]
Red blood cell count (median [IQR])		4.20 [3.77, 4.59]
Platelet count (median [IQR])		225.00 [173.00, 284.00]
PG-SGA (median [IQR])		3.00 [0.00, 7.00]
mGPS (%)	0	2635 (68.7)
	1	775 (20.2)
	2	423 (11.0)
LCS (%)	0	847 (22.1)
	1	2494 (65.1)
	2	492 (12.8)
KPS (median [IQR])		90.00 [80.00, 90.00]
KPS (%)	< 70	172 (4.5)
	≥70	3661 (95.5)
NLR (median [IQR])		2.38 [1.58, 3.76]
PLR (median [IQR])		148.33 [107.73, 211.11]
GLR (median [IQR])		3.64 [2.74, 5.18]
ALI (median [IQR])		37.81 [22.47, 58.56]
SII (median [IQR])		533.33 [313.30, 906.27]
CAR (median [IQR])		0.10 [0.07, 0.40]
GNRI (median [IQR])		101.07 [94.03, 107.86]
mGNRI (median [IQR])		46.80 [41.41, 53.53]
AGR (median [IQR])		1.35 [1.16, 1.54]
PNI (median [IQR])		47.30 [43.20, 51.15]
NRI (median [IQR])		102.28 [95.16, 109.13]
LCR (median [IQR])		3617.02 [880.60, 6788.08]
CONUT (median [IQR])		2.00 [1.00, 3.00]
CONUT (%)	< 2	1708 (44.6)
	≥ 2	2125 (55.4)

AGR, albumin-to-globulin ratio; ALI, advanced lung cancer inflammation index; BMI, body mass index; CAR, C-reactive protein-to-albumin ratio; CONUT score, controlling nutritional status score; CVD: cardiovascular disease; GLR, glucose-to-lymphocyte ratio; GNRI, geriatric nutritional risk index; KPS, Karnofsky performance scoring; LCR, lymphocyte-to-C reactive protein ratio; mGNRI, modified geriatric nutritional risk index; mGPS, modified Glasgow prognostic score; NLR, neutrophil-to-lymphocyte ratio; NRI, nutritional risk index; LCS, lymphocyte-to-C-reactive protein ratio score; NSCLC, non-small cell lung cancer; PG-SGA, Patient-Generated Subjective Global Assessment; PLR, platelet-to-lymphocyte ratio; PNI, prognostic nutritional index; SD, standard deviation; SII, systemic immune-inflammation index.

### Association of inflammation/nutrition-based indicators and OS in chemotherapy patients

**Figure 2:**
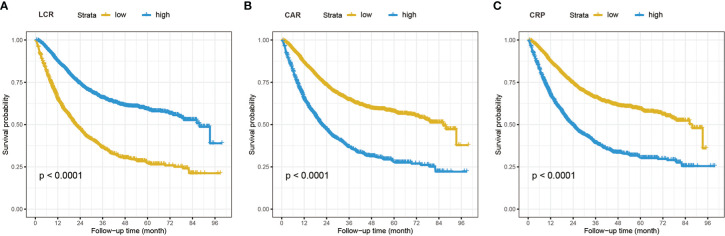
The Kaplan–Meier curves analysis in cancer patients who received chemotherapy of LCR **(A)**, CAR **(B)**, and CRP **(C)**. LCR, lymphocyte-to-CRP ratio; CAR, CRP/albumin ratio; CRP, C-reactive protein.

During the median (IQR) follow-up of 18.9 (10.5, 33.1) months, 1,573 chemotherapy patients died. **Figure S1** shows the crude and adjusted dose-response relationship between 16 inflammation/nutrition-based indicators and OS. Nonlinear and positive relationships were found for all indicators in the crude and adjusted models. The optimal cutoffs of inflammation- and malnutrition-based indicators were 3.29 for NLR, 206.96 for PLR, 5.12 for GLR, 740.70 for SII, 26.42 for ALI, 0.14 for CAR, 93.34 for GNRI, 45.75 for mGNRI, 1.17 for AGR, 46.40 for PNI, 94.41 for NRI, 2812.5 for LCR, and 3.96 for CRP. **Table 2** demonstrates the association of the sixteen indicators with overall survival in chemotherapy patients. All inflammation/nutrition-based indicators were independent risk factors for the survival of chemotherapy patients in the adjusted models when the indicators were assessed as categorical variables. The results from the Kaplan–Meier curves showed that chemotherapy patients with malnutrition or an inflammatory status had worse OS than those without malnutrition or inflammation (**Figures 2** and **S2**).

**Table 2:** The associations of sixteen malnutrition/inflammation-based indicators with overall survival in cancer patients who received chemotherapy in the INSCOC project from 2013-2019.

	Model 1		Model 2		Model 3	
	HR (95% CI)	P value	HR (95% CI)	P value	HR (95% CI)	P value
NLR						
Per SD	1.16 (1.11,1.21)	<0.001	1.10 (1.05,1.16)	<0.001	1.03 (0.99,1.10)	0.078
< 3.29 vs. ≥ 3.29	2.24 (2.03,2.48)	<0.001	1.70 (1.53,1.90)	<0.001	1.47 (1.32,1.65)	<0.001
PLR						
Per SD	1.15 (1.11,1.20)	<0.001	1.10 (1.06,1.15)	<0.001	1.05 (1.01,1.10)	0.014
< 213 vs. ≥ 213	1.68 (1.51,1.87)	<0.001	1.40 (1.25,1.57)	<0.001	1.24 (1.09,1.38)	<0.001
GLR						
Per SD	1.25 (1.04,1.49)	0.017	1.08 (0.89,1.31)	0.455	1.03 (0.84,1.26)	0.733
< 5.12 vs. ≥ 5.12	1.64 (1.47,1.82)	<0.001	1.34 (1.20,1.50)	<0.001	1.32 (1.18,1.49)	<0.001
ALI						
Per SD	0.68 (0.63,0.73)	<0.001	0.79 (0.74,0.86)	<0.001	0.88 (0.82,0.95)	<0.001
< 26.42 vs. ≥ 26.42	0.43 (0.38,0.47)	<0.001	0.59 (0.52,0.65)	<0.001	0.69 (0.62,0.77)	<0.001
SII						
Per SD	1.14 (1.1,1.18)	<0.001	1.09 (1.04,1.14)	<0.001	1.02 (0.98,1.07)	0.223
< 740.7 vs. ≥ 740.7	2.00 (1.81,2.21)	<0.001	1.6 (1.44,1.78)	<0.001	1.34 (1.20,1.51)	<0.001
CAR						
Per SD	1.28 (1.22,1.33)	<0.001	1.19 (1.14,1.25)	<0.001	1.11 (1.05,1.16)	<0.001
< 0.14 vs. ≥ 0.14	2.45 (2.21,2.7)	<0.001	1.83 (1.64,2.04)	<0.001	1.54 (1.38,1.73)	<0.001
GNRI						
Per SD	0.71 (0.67,0.74)	<0.001	0.70 (0.65,0.75)	<0.001	0.76 (0.71,0.81)	<0.001
< 93.3 vs. ≥ 93.3	0.51 (0.45,0.56)	<0.001	0.59 (0.52,0.67)	<0.001	0.67 (0.58,0.76)	<0.001
mGNRI						
Per SD	0.71 (0.67,0.74)	<0.001	0.71 (0.65,0.77)	<0.001	0.78 (0.71,0.84)	<0.001
< 45.7 vs. ≥ 45.7	0.51 (0.45,0.56)	<0.001	0.55 (0.48,0.63)	<0.001	0.62 (0.54,0.71)	<0.001
AGR						
Per SD	0.72 (0.68,0.76)	<0.001	0.79 (0.75,0.84)	<0.001	0.84 (0.79,0.86)	<0.001
< 1.17 vs. ≥ 1.17	0.51 (0.46,0.57)	<0.001	0.63 (0.56,0.7)	<0.001	0.69 (0.61,0.77)	<0.001
PNI						
Per SD	0.69 (0.65,0.72)	<0.001	0.76 (0.72,0.81)	<0.001	0.82 (0.77,0.86)	<0.001
< 46.4 vs. ≥ 46.4	0.52 (0.48,0.58)	<0.001	0.65 (0.58,0.72)	<0.001	0.71 (0.63,0.79)	<0.001
NRI						
Per SD	0.71 (0.67,0.74)	<0.001	0.70 (0.65,0.75)	<0.001	0.75 (0.69,0.80)	<0.001
< 94.4 vs. ≥ 94.4	0.50 (0.45,0.56)	<0.001	0.59 (0.51,0.67)	<0.001	0.66 (0.58,0.76)	<0.001
LCR						
Per SD	0.66 (0.59,0.73)	<0.001	0.77 (0.70,0.85)	<0.001	0.80 (0.73,0.90)	<0.001
< 2812.5 vs. ≥2812.5	0.38 (0.35,0.43)	<0.001	0.52 (0.46,0.58)	<0.001	0.60 (0.53,0.67)	<0.001
CONUT						
As continuous	1.55(1.44,1.66)	<0.001	1.33(1.24,1.49)	<0.001	v1.26(1.16,1.38)	<0.001
< 2 vs. ≥ 2	1.62 (1.46,1.80)	<0.001	1.33(1.19,1.49)	<0.001	1.27(1.14,1.43)	<0.001
CRP						
Per SD	1.26(1.21,1.31)	<0.001	1.19(1.13,1.25)	<0.001	1.11(1.06,1.17)	<0.001
< 3.96 vs. ≥ 3.96	2.37(2.14,2.63)	<0.001	1.81(1.63,2.02)	<0.001	1.55(1.39,1.74)	<0.001
mGPS						
0	Ref.		Ref.		Ref.	
1	2.12(1.89,2.38)	<0.001	1.59(1.41,1.81)	<0.001	1.36(1.20,1.56)	<0.001
2	3.20(2.79,3.68)	<0.001	2.29(1.97,2.65)	<0.001	1.85(1.58,2.16)	<0.001
LCS						
0	Ref.		Ref.		Ref.	
1	2.14(1.86,2.46)	<0.001	1.66(1.43,1.94)	<0.001	1.51(1.29,1.76)	<0.001
2	3.57(3.00,4.24)	<0.001	2.34(1.94,2.82)	<0.001	2.02(1.67,2.46)	<0.001

Model 1: univariate analysis; Model 2: adjusted for age, sex, tumor stage, and BMI (except for ALI); Model 3, adjusted for age, sex tumor stage, BMI (except for ALI), KPS, PG-SGA, surgery, radiotherapy, targeted therapy, immunotherapy, smoking, alcohol drinking, history of hepatitis (cirrhosis), stroke, CVD, hypertension, and diabetes.

AGR, albumin-to-globulin ratio; ALI, advanced lung cancer inflammation index; CONUT score, controlling nutritional status score; CRP, C-reactive protein; GLR, glucose-to-lymphocyte ratio; GNRI, geriatric nutritional risk index; LCR, lymphocyte-to-C-reactive protein ratio; LCS, lymphocyte-to-C-reactive protein ratio score; mGNRI, modified geriatric nutritional risk index; mGPS, modified Glasgow prognostic score; NLR, neutrophil-to-lymphocyte ratio; NRI, nutritional risk index; PLR, platelet-to-lymphocyte ratio; PNI, prognostic nutritional index; SII, neutrophil immune-inflammation index; CAR, C-reactive protein-to-albumin ratio.

### Comparison of the prognostic ability of all indicators

**Figure 3:**
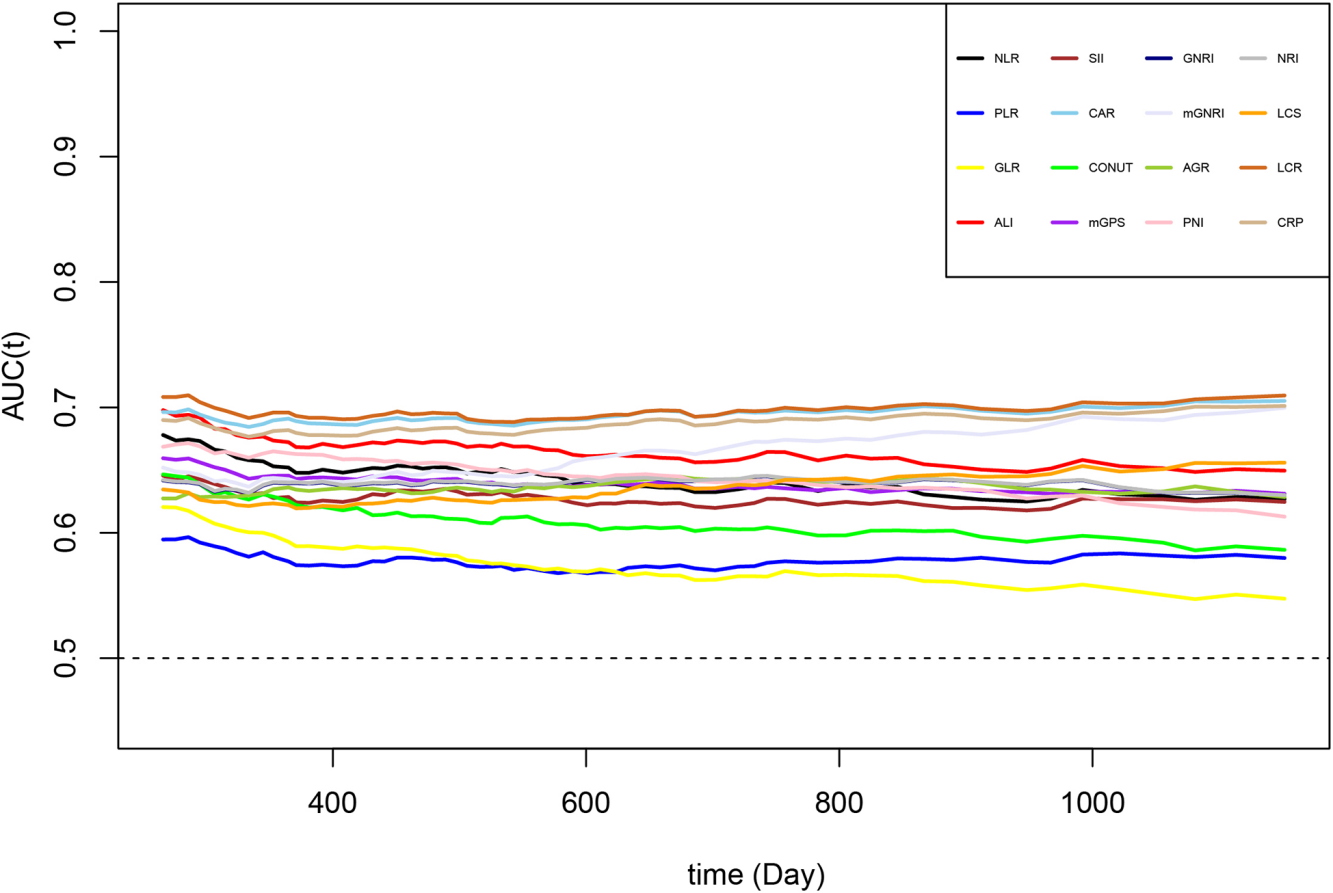
The time-dependent ROC of malnutrition/inflammation-based indicators for diagnosing overall survival in cancer patients who received chemotherapy in the INSCOC project from 2013-2019. AGR, albumin-to-globulin ratio; ALI, advanced lung cancer inflammation index; CAR, neutrophil-to-lymphocyte ratio; CONUT score, controlling nutritional status score; CRP, C-reactive protein; GLR, glucose-to-lymphocyte ratio; GNRI, geriatric nutritional risk index; LCR, lymphocyte-to-C-reactive protein ratio; LCS, lymphocyte-to-C-reactive protein ratio score; mGNRI, modified geriatric nutritional risk index; mGPS, modified Glasgow prognostic score; NLR, neutrophil-to-lymphocyte ratio; NRI, nutritional risk index; PLR, platelet-to-lymphocyte ratio; PNI, prognostic nutritional index; SII, neutrophil immune-inflammation index.

Comparisons of the prognostic ability of 16 inflammation/nutrition-based indicators for the mortality of chemotherapy patients was performed using the time-ROC and ROC curves (**Table 3**, **Figure 3**). Among 16 indicators, the LCR (0.658 [0.644, 0.673]) had the highest C-index for OS of chemotherapy patients, followed by CAR (0.653 [0.639, 0.668]) and CRP (0.647 [0.633, 0.662]). In line with the results of the time-dependent AUC, the LCR had the highest and most stable AUC value, followed by the CAR and CRP during the follow-up period. As expected, the level of inflammation assessed by LCR, CAR, and CRP increased with increasing tumor stage (**Figure S3**). As the top 3 indicators, LCR, CAR, and CRP are all based on CRP levels. We further explored whether the predictive ability of the top 3 indicators reached statistical significance, as shown in **Table S2**. Compared with CRP, LCR had a significantly higher predictive performance. However, no difference was found in the predictive performance between CRP and CAR.

**Table 3:** The C-index of 16 malnutrition/inflammation-based indicators for OS in cancer patients who received chemoradiotherapy in the INSCOC project from 2013-2019.

	C-index	95% CI
LCR	**0.658**	**0.644, 0.673**
CAR	**0.653**	**0.639, 0.668**
CRP	**0.647**	**0.633, 0.662**
ALI	0.642	0.628, 0.657
NLR	0.626	0.611, 0.640
PNI	0.624	0.609, 0.639
mGNRI	0.621	0.606, 0.635
mGPS	0.615	0.602, 0.628
NRI	0.612	0.597, 0.627
GNRI	0.612	0.597, 0.627
SII	0.607	0.592, 0.622
AGR	0.603	0.588, 0.618
LCS	0.599	0.587, 0.612
CONUT	0.598	0.583, 0.613
GLR	0.573	0.558, 0.589
PLR	0.564	0.548, 0.579

AGR, albumin-to-globulin ratio; ALI, advanced lung cancer inflammation index; CAR, C-reactive protein-to-albumin ratio; CONUT score, controlling nutritional status score; GLR, glucose-to-lymphocyte ratio; GNRI, geriatric nutritional risk index; LCR, lymphocyte-to-C reactive protein ratio; mGNRI, modified geriatric nutritional risk index; mGPS, modified Glasgow prognostic score; NLR, neutrophil-to-lymphocyte ratio; NRI, nutritional risk index; LCS, lymphocyte-to-C-reactive protein ratio score; PLR, platelet-to-lymphocyte ratio; PNI, prognostic nutritional index; SII, systemic immune-inflammation index. The top three indicators were presentedin the bold values.

### Subgroup analysis and joint-effect analysis

**Figure 4:**
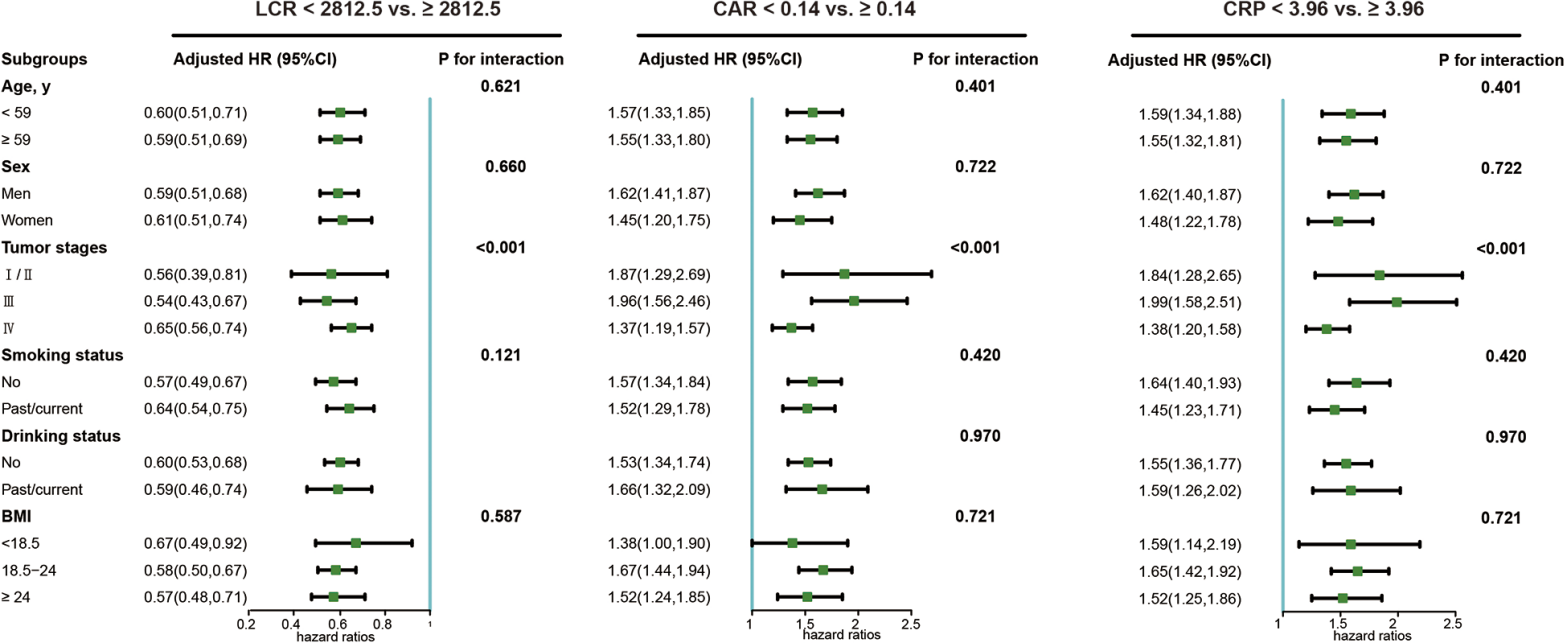
The sub-group analysis of the association of LCR, CRP and CRP with overall survival in cancer patients who received chemotherapy in the INSCOC project from 2013-2019. Models were adjusted for age, sex, tumor stage, BMI (except for ALI), KPS, PG-SGA, surgery, radiotherapy, targeted therapy, immunotherapy, smoking, alcohol drinking, history of hepatitis (cirrhosis), stroke, CVD, hypertension, and diabetes. LCR, lymphocyte-to-CRP ratio; CAR, CRP/albumin ratio; CRP, C-reactive protein.

The LCR, CAR, and CRP were selected as the top three indicators for the survival of chemotherapy patients. The subgroup analyses of LCR, CAR, and CRP with the overall survival of chemotherapy patients are illustrated in **Figure 4**. A significant association between inflammatory status and worse survival outcomes was found in all subgroups when chemotherapy patients were stratified by age, tumor stage, BMI, drinking, and smoking status. Notably, the tumor stage significantly modified the association between inflammatory status and worse survival outcomes (P for interaction < 0.05). We further divided the study population into four groups based on the absence/presence of elevated LCR levels (optimal cutoff) and tumor stages (I/II vs. III/IV). Chemotherapy patients with low LCR and III/IV tumor stages exhibited the worst overall survival compared with patients with high LCR and I/II tumor stages (**Figure S4**). In addition, patients with low LCR and III/IV tumor stages were associated with a 6-fold increased risk of death (HR=5.92, 95% CI: 4.58, 7.66) compared with patients with high LCR and I/II tumor stages in the multivariate analysis (**Table 4**).

**Table 4:** The joint effect of LCR and tumor stage on the survival in cancer patients who received chemotherapy in the INSCOC project from 2013-2019.

	Crude-models		Adjusted-models	
	HR (95%CI)	p-value	HR (95%CI)	p-value
High LCR+Stage I/II	Ref.	Ref.		
Low LCR+Stage I/II	2.76 (1.97, 3.87)	<0.001	2.25 (1.58,3.18)	<0.001
High LCR+Stage III/IV	4.35 (3.41, 5.56)	<0.001	3.42 (2.65,4.42)	<0.001
Low LCR+Stage III/IV	9.83 (7.73, 12.51)	<0.001	5.92 (4.58,7.66)	<0.001

Adjusted models included age, sex, BMI, KPS, PG-SGA, surgery, radiotherapy, targeted therapy, immunotherapy, smoking, alcohol drinking, history of hepatitis (cirrhosis), stroke, CVD, hypertension, and diabetes.

The cutoff of LCR was 2812.5.

## Discussion

Evidence has shown that inflammatory/nutrition-based indicators are reliable predictors of the outcomes for patients with cancer; however, the optimal choice of various indicators is still unclear. In this multicenter, prospective cohort study, we found that LCR, CAR, and CRP were the top three indicators for the prediction of the outcomes of chemotherapy patients. In addition, the LCR had the best prognostic ability among 16 inflammatory/nutrition-based indicators. We also observed a significant interaction between inflammation (assessed by LCR, CAR, and CRP), tumor stage, and overall survival.

The lymphocyte–C-reactive protein ratio (LCR) is the combination of lymphocyte count and CRP, and its prognostic value has been explored in several previous studies. By analyzing 1,303 patients with stage II/III colon cancer who received surgery, Suzuki S et al. investigated 16 inflammation-related markers and found that LCR (≤ 12,980) was most significantly correlated with worse overall survival (OS) and disease-free survival (DFS) (10). By analyzing 477 colorectal cancer patients from the discovery (n = 373) and validation cohorts (n=104), Okugawa Y et al. concluded that preoperative LCR is a useful marker for the perioperative and postoperative management of colorectal cancer patients. They also found that a low LCR was also associated with postoperative infectious complications, indicating the role of the LCR in predicting both short- and long-term postoperative outcomes of CRC patients (11).

CAR is also a sensitive biomarker for cancer-related prognosis. By analyzing 133 patients with stage III colorectal cancer, Matsuoka H et al. found that postoperative CAR (≥ 0.035) is strongly associated with a poor prognosis and was useful for deciding whether adjuvant chemotherapy should be used (12). In a retrospective study, Ide S et al. evaluated the association between CAR and prognosis in 115 rectal cancer patients and found that CAR ≥ 0.049 before neoadjuvant chemoradiotherapy was an independent prognostic factor for OS and DFS (13). Dolan RD et al. reported that CAR> 0.22 was an independent predictor for the OS of CRC patients (14).

During the preoperative or postoperative period, CRP is one of the most useful parameters for evaluating the degree of inflammation in cancer patients. By drawing data from 300 CRC patients, Koike Y et al. reported that preoperative CRP was a prognostic variable in patients with stage I/II CRC. Another study including 74 lymph node-positive upper tract urothelial carcinoma patients found that preoperative CRP levels were significantly associated with poor survival in the multivariate analysis. Notably, most previous studies adopted CRP-related markers that were collected before the operation. Additionally, in the general clinical setting, CRP is not a routine test, which limits the extrapolation of those markers. Another study conducted in Japan found that postoperative (but not preoperative) indicators, including CAR, LCR, and NLR, were significantly associated with worse overall survival (15).

CRP-related markers, including LCR, CAR, and CRP, have the best predictive performance in chemotherapy patients. We also explored the effect of other indicators on overall survival. The positive associations between other inflammatory/nutrition-based indicators and OS were in line with findings from previous studies. Based on previous studies (16–18), NLR is a powerful biomarker that predicts prognosis in various types of cancer with cutoff values ranging from 2 to 5, depending on the study population and the method of definition of the cutoff value. GPS is also useful for the prediction of prognosis. Notably, modified GPS (mGPS) has proven useful in predicting the postoperative and preoperative prognosis of CRC patients undergoing curative surgery (19, 20). Similarly, several previous studies also explored the usefulness of other albumin-related prognostic markers, including PNI, ALI, and AGR, for the prognosis of cancer patients (21–23). Platelet-related markers, including PLR and SII, were also evaluated as independent predictors for poor OS in cancer patients (24, 25).

To implement preventive analytic strategies, it is crucial to develop prognostic models that estimate risk as accurately as possible. In the current study, the top 3 prognostic indicators were all CRP-related indicators with minor differences in the value of the C-index. CRP is a more convenient and cost-effective indicator than LCR and CAR, and it is important to know whether the difference in the predictive value is statistically significant. The results from both NRI and IDI showed that the predictive ability of LCR significantly outperformed that of CRP, demonstrating the importance of LCR as the best option to predict the prognosis of chemotherapy patients (**Table S2**).

The tumor stage significantly modified the association between CRP-related indicators and the prognosis of chemotherapy patients. We found that chemotherapy patients with advanced tumor stages (III/IV) and a high inflammatory burden exhibited a 6-fold higher risk of death compared with patients with early tumor stages and low levels of inflammation. As a recognized hallmark of cancer, inflammation plays a substantial role in the development and progression of cancer (26). On the other hand, solid cancers can induce an inflammatory microenvironment. In short, special attention should be given to patients with advanced tumor stages and a high inflammatory burden.

The main strength of the current study was that it first found that the LCR was the best predictor of prognosis for chemotherapy patients among the 16 metrics presented in a large, population-based, multicenter cohort study. In addition, this study fully considered the effects of potential confounding factors, such as lifestyle habits and laboratory measurements. Finally, the strengths of this study also include its prospective study design, large sample size, long-term follow-up, and subgroup analysis.

Limitations should also be noted in the current study. First, due to the limited data on targeted therapy, immunotherapy and the use of anti-inflammatory drugs, including aspirin and statins, the effect of these confounders could not be elucidated. Second, data on the 16 inflammatory/nutrition-based indicators were collected only once, and further studies should be conducted to better explore the impact of long-term patterns of inflammatory/nutrition-based indicators on the survival of chemotherapy patients. Third, we used only preoperative data, not postoperative data, and whether the predictive value changes during the operation needs to be explored in future studies. Fourth, due to the limited data, the prognostic values of other inflammation-based indicator can’t be explored in the current study. For example, the ratio of fibrinogen to pre-albumin (FPR) as well as its combination with SII were found to be superior to other inflammatory biomarkers for predicting the overall survival among patients with advanced NSCLC (27) or early recurrence among stage II-III colorectal cancer patients after curable resection (28). However, the INSCOC project has no data on the levels of fibrinogen, and whether FPR outperformed in predicting survival in chemotherapy patients with cancer should be better elucidated in future studies.

## Conclusions

The LCR, CAR, and CRP were the top three predictors for the prognosis of chemotherapy patients in clinical practice, and the predictive ability of the LCR significantly outperformed that of the CRP. In addition, chemotherapy patients with advanced tumor stage and high inflammatory burden had a significantly higher risk of death than patients with early tumor stage and low levels of inflammation.

## Data availability statement

The raw data supporting the conclusions of this article will be made available by the authors, without undue reservation.

## Ethics statement

This study followed the Helsinki Declaration. All participants signed informed consent forms, and this study was approved by the Institutional Review Board of each hospital (Registration number: ChiCTR1800020329). The patients/participants provided their written informed consent to participate in this study.

## Author contributions

TL: methodology, software, writing-original draft preparation; CL: visualization; LD: methodology, software; MS: resources; SL: supervision, validation; HS: conceptualization, supervision, validation, resources. All authors contributed to the article and approved the submitted version.
