# Supplementary material for: The prognostic effect of sixteen malnutrition/inflammation-based indicators on the overall survival of chemotherapy patients
Source: Front Immunol. 2023 Feb 16;14:1117232. doi: 10.3389/fimmu.2023.1117232 (PMC9978470; doi:10.3389/fimmu.2023.1117232)
Supplement: Supplementary file 1 [file DataSheet_1.docx]

**Supplementary Materials**

**Table S1.** **Calculation methods of each nutrition/inflammation-based indicator.**

| **Indicators** | **Definition or calculation formula** | |
| --- | --- | --- |
| **ALI** | BMI (kg/m^2^) × albumin(g/dl)/NLR (×10^9^) | |
| **SII** | platelet count (×10^9^) × neutrophil count (×10^9^)/lymphocyte count (×10^9^) | |
| **PLR** | platelet count (×10^9^)/lymphocyte count (×10^9^) | |
| **NLR** | neutrophil count (×10^9^)/ lymphocyte count (×10^9^) | |
| **PNI** | albumin (g/L) + 5×lymphocyte count (×10^9^) | |
| **CAR** | C-reactive protein (mg/ L)/ albumin (g/ L) | |
| **GLR** | glucose (mmol/l)/ lymphocyte count (×10^9^) | |
| **LCR** | 10,000×lymphocyte count (×10^9^)/CRP (mg/L) | |
| **AGR** | albumin (g/L)/globulin (g/L) | |
| **GNRI** | 1.489×albumin (g/L) + (41.7× current weight/ ideal body weight (IBW)) | |
| **mGNRI** | 14.89/CRP(mg/L)+41.7×current weight/ IBW | |
| **NRI** | 1.519× albumin (g/L) + 41.7× current weight/ IBW | |
| **mGPS** |  | |
| Score 0 | CRP (≤10 mg/L) | |
| Score 1 | CRP (>10 mg/L) and albumin (≥35 g/L) | |
| Score 2 | CRP (>10 mg/L) and albumin (<35 g/L) | |
| **LCS** |  | |
| Score 0 | lymphocyte count ≥1×10^9^/L and CRP ≤3.0 mg/L | |
| Score 1 | lymphocyte count <1×10^9^/L and CRP ≤3.0 mg/L | |
| Score 1 | lymphocyte count ≥1×10^9^/L and CRP >3.0 mg/L | |
| Score 2 | lymphocyte count <1×10^9^/L and CRP >3.0 mg/L | |
| **CONUT score** | |  |
| Serum albumin (g/dL) | | albumin score |
| 3.5-4.5 | | 1 |
| 30-34.9 | | 2 |
| 25-29.9 | | 4 |
| <25 | | 6 |
| Total lymphocyte (count/mm3) | | TLC score |
| ≥1600 | | 0 |
| 1200-1599 | | 1 |
| 800-1199 | | 2 |
| 800 | | 3 |
| Total cholesterol (mg/dL) | | TC score |
| ≥180 | | 0 |
| 140-180 | | 1 |
| 100-139 | | 2 |
| 100 | | 3 |
| CONUT score (total) | | albumin score+ TLC score+ TC score |

Note: IBW: ideal body weight; If current weight is greater than IBW, current/IBW is regarded as 1. The IBW of men and women were calculated by the formula, height(cm)-100-[height (cm)-150/4] and height(cm)-100-[height (cm)-150/2.5], respectively.

**Table S2. The comparison of the predictive value of CRP, CAR and LCR**

|  | **C-index** | |  | **IDI** | |  | **NRI** | |
| --- | --- | --- | --- | --- | --- | --- | --- | --- |
|  | **Value** | **95% CI** |  | **Difference** | **p-value** |  | **Difference** | **p-value** |
| **LCR** | 0.658 | 0.644, 0.673 |  | 0.070(0.042,0.099) | <0.001 |  | 0.238(0.167,0.309) | <0.001 |
| **CAR** | 0.653 | 0.639, 0.668 |  | 0(-0.002,0.003) | 0.939 |  | -0.159(-0.219,0.140) | 0.172 |
| **CRP** | 0.647 | 0.633, 0.662 |  | Ref. |  |  | Ref. |  |

Note: CAR, C-reactive protein-to-albumin ratio; LCR, lymphocyte-to-C reactive protein ratio; CRP, C-reactive protein. NRI, net reclassification index; IDI: integrated discrimination improvement.


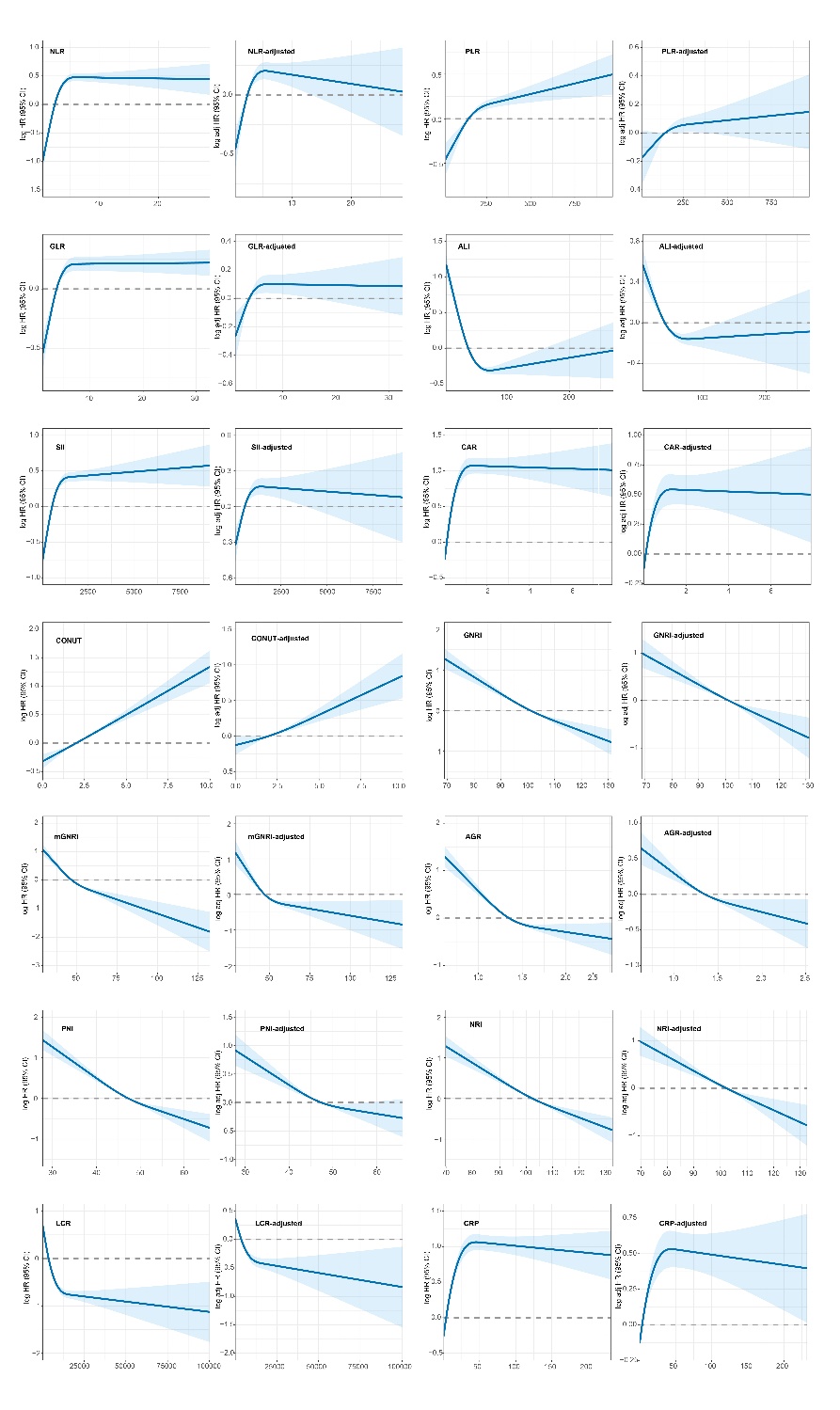


**Figure S1. The unadjusted and adjusted restricted cubic spline of other malnutrition/ inflammation-based indicators in chemotherapy patients.**

Note: Models were adjusted for age, sex, tumor stage, BMI (except for ALI), KPS, PG-SGA, surgery, radiotherapy, targeted therapy, immunotherapy, smoking, and alcohol drinking. AGR: albumin-to-globulin ratio; ALI: advanced lung cancer inflammation index; CONUT score: controlling nutritional status score; GLR: glucose-to-lymphocyte ratio; GNRI: geriatric nutritional risk index; LCS: lymphocyte-to-C-reactive protein ratio score; mGNRI: modified geriatric nutritional risk index; mGPS: modified Glasgow prognostic score; NLR: neutrophil-to-lymphocyte ratio; NRI: nutritional risk index; PLR: platelet-to-lymphocyte ratio; PNI: prognostic nutritional index; SII: neutrophil immune-inflammation index.


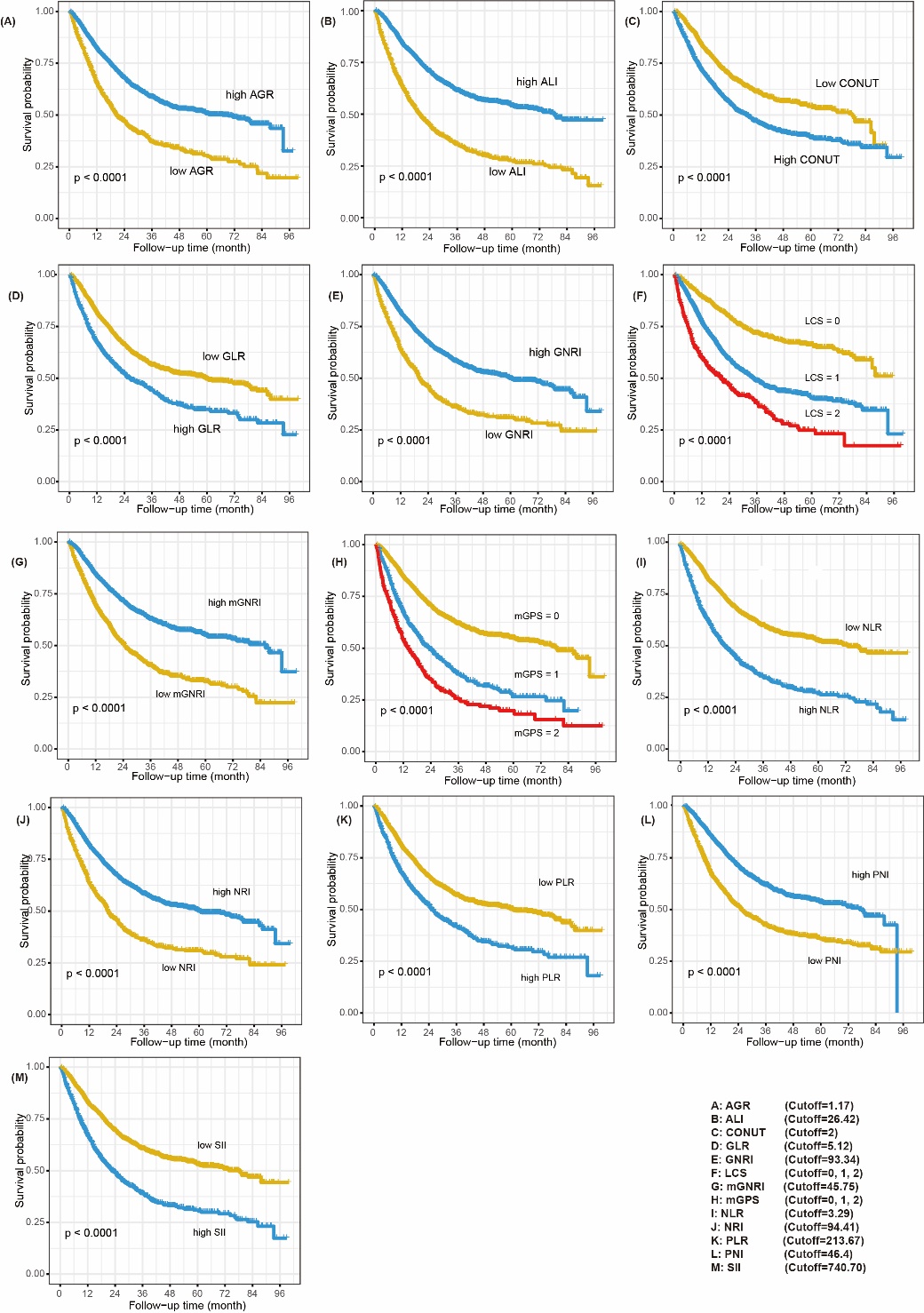


**Figure S2.** **The Kaplan-–Meier curves of the other thirteen indicators in chemotherapy patients.**

Note: Models were adjusted for age, sex, tumor stage, BMI (except for ALI), KPS, PG-SGA, surgery, radiotherapy, smoking, and alcohol drinking. AGR: albumin-to-globulin ratio; ALI: advanced lung cancer inflammation index; CONUT score: controlling nutritional status score; GLR: glucose-to-lymphocyte ratio; GNRI: geriatric nutritional risk index; LCS: lymphocyte-to-C-reactive protein ratio score; mGNRI: modified geriatric nutritional risk index; mGPS: modified Glasgow prognostic score; NLR: neutrophil-to-lymphocyte ratio; NRI: nutritional risk index; PLR: platelet-to-lymphocyte ratio; PNI: prognostic nutritional index; SII: neutrophil immune-inflammation index.


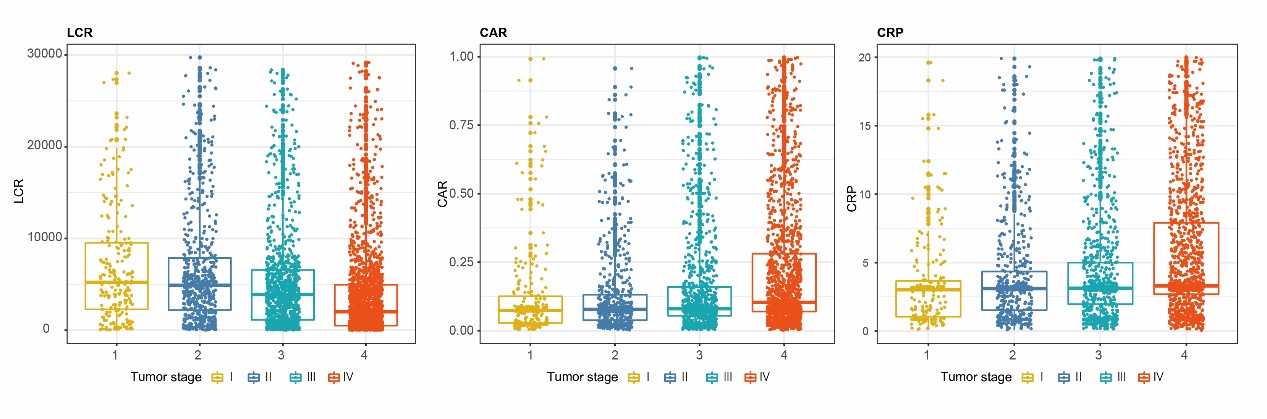
 **Figure S3. The scatterplot of LCR, CAR and CRP in different TNM stages.**

LCR: lymphocyte-to-CRP ratio; CAR: CRP/albumin ratio; CRP: C-reactive protein.


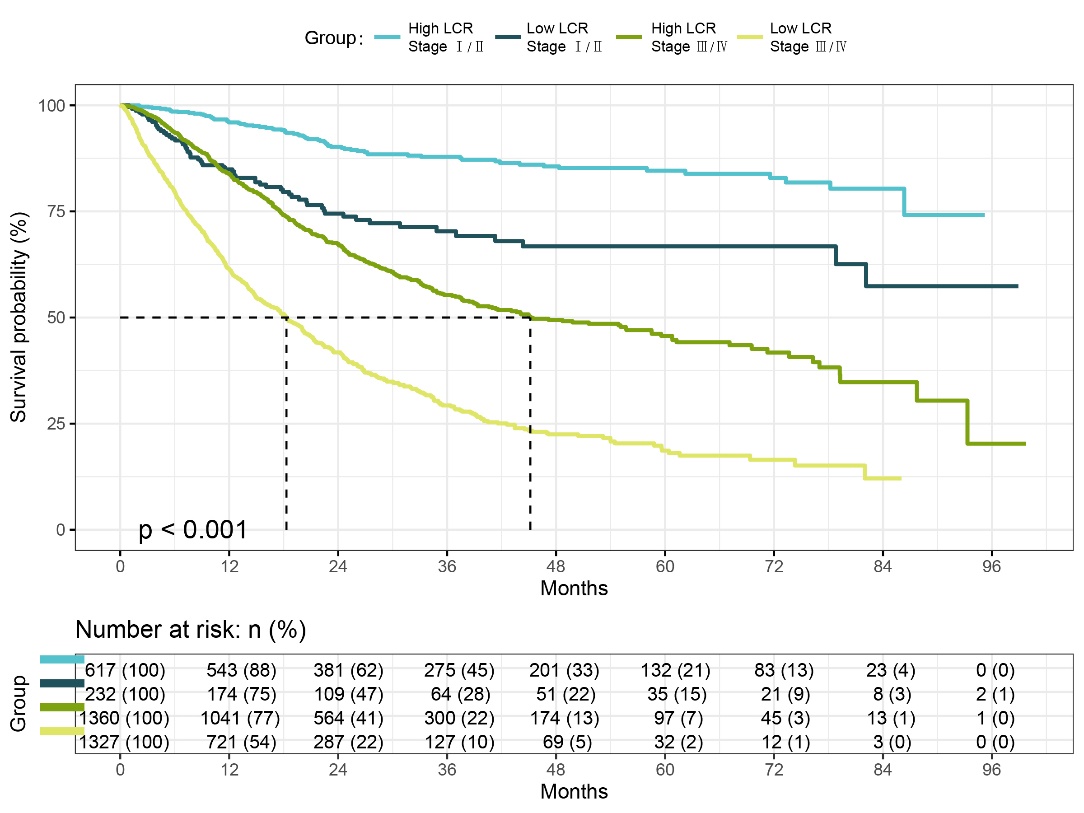


**Figure S4. The Kaplan-–Meier curves of the presence/absence of malnutrition and inflammation in chemotherapy patients.**
